# Re-calibration of the magnetic compass in hand-raised European robins (*Erithacus rubecula*)

**DOI:** 10.1038/srep14323

**Published:** 2015-09-21

**Authors:** Bianca Alert, Andreas Michalik, Nadine Thiele, Michael Bottesch, Henrik Mouritsen

**Affiliations:** 1 Institut für Biologie und Umweltwissenschaften, Carl von Ossietzky Universität Oldenburg, D-26111 Oldenburg, Germany; 2 Research Centre for Neurosensory Sciences, University of Oldenburg, Carl-von-Ossietzky-Straße 9-11, D-26129 Oldenburg, Germany

## Abstract

Migratory birds can use a variety of environmental cues for orientation. A primary calibration between the celestial and magnetic compasses seems to be fundamental prior to a bird’s first autumn migration. Releasing hand-raised or rescued young birds back into the wild might therefore be a problem because they might not have established a functional orientation system during their first calendar year. Here, we test whether hand-raised European robins that did not develop any functional compass before or during their first autumn migration could relearn to orient if they were exposed to natural celestial cues during the subsequent winter and spring. When tested in the geomagnetic field without access to celestial cues, these birds could orient in their species-specific spring migratory direction. In contrast, control birds that were deprived of any natural celestial cues throughout remained unable to orient. Our experiments suggest that European robins are still capable of establishing a functional orientation system after their first autumn. Although the external reference remains speculative, most likely, natural celestial cues enabled our birds to calibrate their magnetic compass. Our data suggest that avian compass systems are more flexible than previously believed and have implications for the release of hand-reared migratory birds.

To orient during their extensive annual journeys towards their wintering quarters and back to their breeding grounds, migratory birds possess several independently working compass systems. They can use the geomagnetic field^1^,^2^,^3^, and celestial cues such as the stars^4^,^5^,^6^, the sun^7^,^8^ and maybe polarised light patterns^9^,^10^,^11^ as directional cues for compass orientation. These compasses work independently from each other^12^,^13^.

The variety of exploited orientation cues has led many researchers to ask whether birds pool and integrate directional information from their independently working compass systems or if information from the different compass systems is weighed hierarchically to decide for one migratory direction^13^,^14^,^15^. Cue-conflict experiments with migrating birds so far revealed rather ambiguous relations between celestial and geomagnetic orientation cues^16^,^17^. Studies mainly performed in North America report the birds’ magnetic compass to be frequently calibrated by a celestial reference^2^,^11^,^18^ whereas other studies mainly performed with European migratory songbirds report the magnetic compass to be dominant upon or unaffected by conflicting information from celestial cues^19^,^20^.

Whereas the interactions between the different compass systems during migration appear to be rather complex, the relations seem to be more clear during the juvenile phase of night-migratory songbirds. During ontogeny, the magnetic compass can be calibrated by celestial cues^21^,^22^,^23^,^24^,^25^,^26^,^27^,^28^. Juvenile birds have to learn the celestial compasses^4^,^6^,^29^. To establish a functional star compass, naïve birds have to detect the centre of celestial rotation, which coincides with the Polar Star in the northern hemisphere, and to interpret this as “North”^4^. Consecutively, the birds can learn obvious star patterns around the rotational axis^5^,^30^. This enables birds to orient by their star compass during migration even without perceiving celestial rotation^4^,^6^,^30^. To establish the sun compass, young homing pigeons have to observe the path of the sun during different times of day and link its positions to their internal clock^29^.

Many learning processes during ontogeny involve imprinting mechanisms relying on predisposed responses to key stimuli during a sensitive period^31^,^32^. Indeed, there is evidence for a sensitive period in both star^4^,^5^,^24^ and sun compass learning^29^. But whereas the sun compass of homing pigeons appears to be rather flexible even after full development^29^, the star compass of night-migratory songbirds seems to be fixed once it has been learned^5^. Emlen’s hand-raised indigo buntings, *Passerina cyanea*, learned to associate celestial north with a “wrong” rotational centre of the naturally occurring star patterns simulated by a planetarium sky, and they failed to re-orient to the natural sky rotating around the “real” celestial north even after being housed in an outdoor aviary with full view of all celestial cues for six months^5^.

Hence, if juvenile night-migratory songbirds did not have the opportunity to observe any celestial rotation before the onset of their first migration, the calibration process of their magnetic compass might not take place. Although a species-specific magnetic compass direction seems to be inherited in some migratory bird species^33^,^34^,^35^, hand-raised birds prevented from observing celestial rotation often show orientation difficulties during their first migratory period^4^,^5^,^24^. Also, celestial rotation provides a potentially important polarity cue for magnetic compass orientation of birds bred at higher geographic latitudes with steep angles of magnetic inclination^22^,^23^,^36^,^37^. Thus, it appears that it might not always be the case that the inherent magnetic compass alone can be sufficient for appropriate migratory orientation. It might therefore be essential for migratory birds to adjust their magnetic compass to an external geographic reference^14^,^24^.

However, hand-raising and care of rescued fledgling migratory birds often take place indoors without access to natural orientation cues. The main motivation for the present study was to answer the following key question: Can migratory birds which did not develop a functional orientation system before or during their first autumn migration develop it later and thus survive after release back into the wild? That this might work is suggested by a study in which 20 hand-raised blackcaps were released in June as one year old birds around Radolfzell, Germany. One of these birds returned to breed around the release site the following year^38^. However, we do not know whether this bird performed long-distance migration or stayed in the vicinity of the release site. Thus, to answer the key question posed above, experimental evidence is needed. We therefore exposed hand-raised migratory European robins, *Erithacus rubecula*, which had not experienced useful celestial cues during their first half year and which were thus unable to orient in their appropriate migratory direction during their first autumn migration with their magnetic compass, to natural celestial cues during late winter and early spring in an outdoor aviary. During their consecutive spring migration period, we tested whether this delayed exposure to celestial cues allowed them to acquire a functional magnetic compass.

## Results

**Figure 1:**
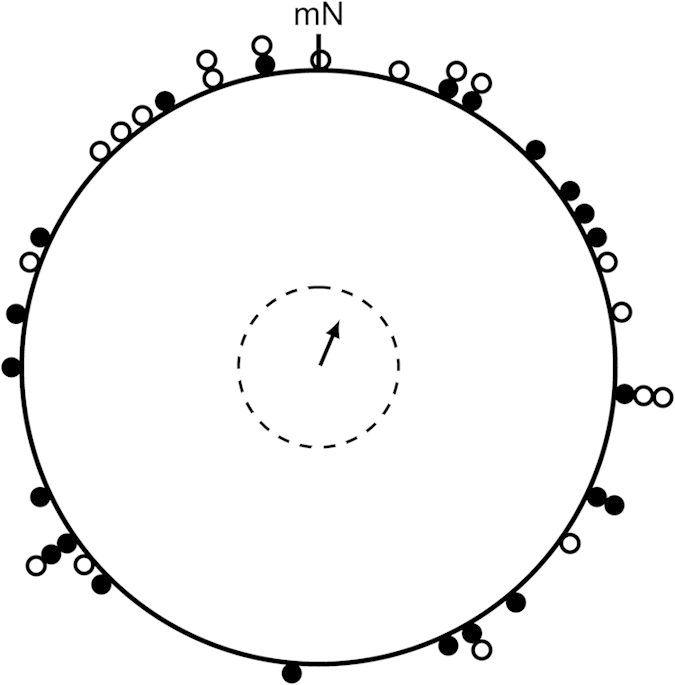
First autumn orientation data (seasons 2010, 2011 and 2013) of all birds when they were tested in the normal magnetic field without access to visual cues. The birds had not developed a functional magnetic compass during their first autumn (mean = 23°, r = 0.17, n = 41, *P* = 0.295). Black dots denote individual mean headings of the birds, which later became the experimental group; open circles denote individual mean headings of the birds, which later were part of the control group. The arrow indicates the overall group mean orientation direction relative to magnetic North (mN). The dotted circle indicates the mean vector length required for 5% significance according to the Rayleigh test.

The hand-raised European robins we used for the experimental part of the present study did not show a significant orientation response during their first individual autumn migratory season when they were tested for their magnetic orientation in the absence of visible celestial cues (Rayleigh test: 2010: mean = 43°, r = 0.32, n = 13, *P* = 0.271; 2011: mean = 69°, r = 0.21, n = 13, *P* = 0.559; 2013: mean = 313°, r = 0.24, n = 15, *P* = 0.418; Total: mean = 23°, r = 0.17, n = 41, *P* = 0.295; Table 1, Fig. 1). Furthermore, their slight tendency to orient towards the north did not correspond to the appropriate species-specific south-westerly autumn migratory direction (*V* test (213°): mean = 23°, *V* = −0.17, *P* = 0.938; in the methods section, the mean species-specific migratory axis for European robins migrating through Lower Saxony in Germany is calculated to be 33–213° based on ringing recoveries). Thus, the birds had not developed a functional magnetic compass during their first autumn.

**Figure 2:**
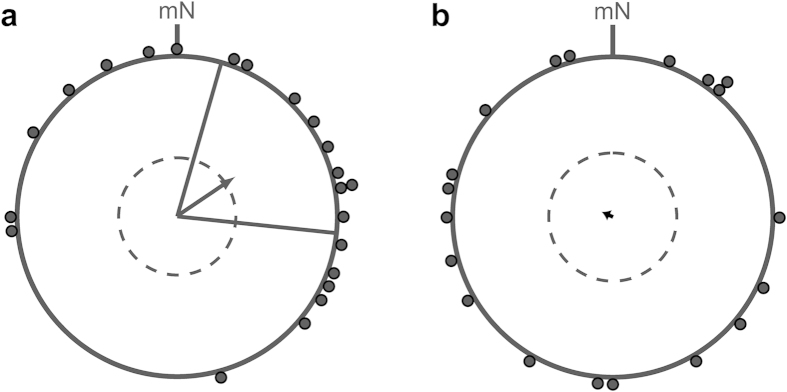
Pooled spring orientation data (seasons 2011, 2012 and 2014) of birds tested in the normal magnetic field without access to visual cues. The experimental birds (**a**) which had been exposed to natural celestial cues from February onwards, oriented significantly in their species-specific north-easterly spring migratory direction (mean = 56° ± 40°, r = 0.43, n = 22, *P* = 0.017) whereas the control birds (**b**) which remained indoors throughout, did not show a directed orientation response (mean = 294°, r = 0.08, n = 19, *P* = 0.895). The arrows represent the group mean orientation directions relative to magnetic North (mN) and the black dots represent the mean orientation directions of individual birds. The dotted circles indicate the mean vector length required for 5% significance according to the Rayleigh test and the solid lines in a indicate the 95% confidence intervals of the group mean orientation vector.

In their following spring migratory season, the experimental group was housed in an aviary from late February onwards. These birds significantly oriented in their species-specific north-easterly spring migratory direction (Rayleigh test: mean = 56°, r = 0.43, n = 22, *P* = 0.004; *V* test (33°): *V* = 0.391, *P* = 0.004; Table 1, Fig. 2a) with the 95% confidence intervals of the mean orientation vector including the expected orientation direction (bootstrapping confidence limits: 14°–95°). In contrast, the control birds that were kept indoors remained disoriented (Rayleigh test: mean = 294°, r = 0.08, n = 19, *P* = 0.895; *V* test (33°): *V* = −0.012, *P* = 0.529; Table 1, Fig. 2b). In single years, the birds of the experimental group were in fact significantly oriented according to the *V* test in 2011 and in 2014 despite the low yearly n (Table 1). In 2012, the birds also showed an orientation tendency towards the same correct migratory direction. The fact that we saw a very similar result in three different years strongly increases our confidence in our main conclusion. Furthermore, a Watson-Williams F test for uniformity of circular data revealed that the means of the individual orientation angles of the experimental birds did not differ significantly between the three seasons (F = 1.307, df = 2, *P* = 0.294).

The pooled orientation data over all seasons of the experimental and control groups, respectively, are depicted in Fig. 2. The orientation of the control group (r = 0.08) was significantly more scattered than the orientation of the experimental group (r = 0.43, *P* = 0.002; 99% confidence limits calculated to be 0.11 < r < 0.75 by bootstrapping [taking 19 values randomly with replacement 100,000 times from the oriented sample in Fig. 2a]). Thus, the concentrations/directedness of the oriented experimental group and of the disoriented control group differed significantly. If considered the other way around, the concentration of the experimental group was also significantly higher than that of the control group (95% confidence limits of the control group’s r = 0.08 calculated to be 0.037 < r < 0.043, *P* = 0.05).

## Discussion

We exposed hand-raised European robins that could not exhibit appropriate magnetic compass orientation during their first autumn migration to natural cues in an outdoor aviary in late winter and early spring. When tested for their magnetic compass orientation during the consecutive spring migratory period, those birds were now able to orient in their seasonally appropriate direction by means of their magnetic compass. In contrast, control birds that remained entirely indoors and thus were never allowed to experience the natural sky continued to show disoriented behaviour when tested for their magnetic compass orientation capabilities.

These results do not support a re-gained dominance of an inherent magnetic compass preference direction as suggested by Able and Able^39^. Able and Able observed that both juvenile and experienced adult Savannah sparrows, *Passerculus sandwichensis*, no longer used the magnetic compass calibration made during autumn when they started their next spring migration despite the fact that they were kept in a windowless room^39^. In contrast to our hand-raised birds, Able and Able worked with wild-caught birds, which were supposed to already have established a functioning magnetic compass prior to the experiments^39^. The birds used in our study only had access to either no or nonsense artificial celestial rotation. They neither exhibited their inherent magnetic preference direction^24^ nor could they use the rotational information to calibrate their magnetic compass accordingly as suggested e.g. by Able and Able^21^. Therefore, the results of Able and Able^39^ do not necessarily contradict our findings but suggest that the birds have a quite flexible orientation system in which a magnetic compass calibration gained during migration can be abolished if the calibrating cue is no longer present.

The apparent re-orientation of our experimental birds relative to the magnetic field implies a re-calibration of their magnetic compass during exposure to natural calibration cues. Even though the calibrating reference cue for the experimental birds in our study remains somewhat speculative, because they have had full access to all natural celestial and magnetic cues, our results support previous studies in which a re-calibration of the magnetic compass relative to conflicting celestial information during the migratory period was observed^2^,^11^.

The considerable scatter in our orientation data might reflect some difficulties of our hand-raised birds in their re-calibration process. However, here one should be particularly careful: it is normal that orientation tests in funnels result in significantly more variability in the orientation responses than seen in the ringing-recovery directions of free-flying wild birds. The main reason for this is that the birds’ motivation to migrate is impossible to determine in caged migratory birds. In nature, night-migratory songbirds typically only migrate once every two or three days^40^. The funnel orientation data are therefore always composed of a mixture of an individual’s highly directed orientation responses on motivated nights and more or less randomly directed responses on less motivated nights. This is usually the main source of the observed scatter in orientation tests in Emlen funnels.

Emlen^4^,^5^ suggested that the birds’ sensitive period for star compass learning is limited to the first pre-migratory period of juvenile birds. Hand-raised birds that were prevented from experiencing celestial rotation until the beginning of their first autumn migration were not able to establish a functioning star compass^4^,^5^. Additionally, Emlen proposed that the star compass could not be re-learned^5^. Some of his birds which had learned an incorrect celestial rotational axis did not refer to the proper rotational axis in the following year despite having had access to natural celestial rotation during the whole summer, but kept orienting by their learnt “north” star^5^. Emlen’s birds were indigo buntings and ours were European robins and even though the basic orientation mechanisms are probably identical in most night-migratory songbird species, the exact hierarchy and/or calibration rules may be species-specific^16^,^17^,^20^,^41^. Thus, species differences could potentially explain the differences between Emlen’s and our results. However, it is much more likely that the important difference between our birds and those tested by Emlen is that Emlen’s birds had learned a functional star compass during their first summer^4^,^5^ whereas our birds had not. Thus, our data when considered in combination with Emlen’s^5^ data suggest that birds can recalibrate their magnetic compass according to natural celestial cues after their first summer if they have not already established a star compass previously. In contrast, night-migratory songbirds seem unable to recalibrate their star and thus magnetic compass according to natural celestial cues after their first summer if they had previously established a wrong star compass.

Our results, when combined with the knowledge in the cited literature, entail two important consequences for migratory birds. First, in some ecological situations, migrating birds should frequently calibrate their different compass systems to a common reference in order to prevent navigational errors^2^,^11^,^42^ and should gather directional information from multiple sources^14^,^15^,^43^. To achieve this, the relative importance of the different cues they use in their orientation system seems to be highly flexible. Secondly, hand-raised birds should be given full access to natural cues as soon as possible to avoid incorrect establishment and/or calibration of their navigational systems during early life. Even if exposure to natural cues has not occurred during their first calendar year, those birds can however still reorient after release. Even in adult birds, there seems to be sufficient plasticity in their orientation system to appropriately respond to current environmental conditions. Thus, hand-raised migratory birds and injured birds kept for longer periods without access to celestial cues still have a good chance of being capable of proper migration after being released. Thus, we recommend releasing such birds back into the wild rather than euthanising such otherwise healthy birds.

## Methods

### Test birds

41 European robin, *Erithacus rubecula*, nestlings were collected for hand-raising from their nests in the vicinity of Oldenburg (Lower Saxony, Germany) during the breeding-time 2010 (13 individuals), 2011 (13 individuals) and 2013 (15 individuals) at the ages between five and eleven days. After they became self-sufficient, the birds were transferred into individual home cages with food and water supplied *ad libitum*. The food was a standardised diet consisting of 28.2% proteins (dried insects and casein), 20% fat (plant oil) and 10% digestible carbohydrates^44^ together with about 8 live mealworms given to each bird daily. The birds’ body weight, fat and moult status were checked weekly. The birds were always kept in a window-less room with no access to natural celestial cues such as the sun, stars or polarised light patterns. The birds could perceive the local geomagnetic field and the local photoperiod, which was mimicked automatically by daylight lamps. All experiments were performed in accordance with EU and national guidelines for the use of animals in research and were approved by local authorities (Niedersächsisches Landesamt für Verbraucherschutz und Lebensmittelsicherheit/LAVES, Germany, license number: AZ 33.9-42502-12-10/0073).

### Early summer experience and autumn experiments

During their first summer, the birds were allowed to experience artificial celestial rotation by an artificial starry sky^6^,^24^,^45^ consisting of LED light diodes of varying modes: either stationary, or rotating or jumping by an average rotational velocity of 15° h^−1^. After the birds had completed their post-juvenile moult and autumn migration had started, it was tested whether they had established a functioning celestial compass from their early experience with the artificial celestial stimulation and/or if they showed an innate magnetic compass orientation response. The detailed orientation data of the autumn migratory season 2010 are published elsewhere^24^. All birds used in this present study were from groups of birds which did not show appropriately directed celestial and/or magnetic orientation during their first autumn migration. At the end of the migratory season, these birds were wintered in their home cages in the same window-less animal room. At the end of February, approximately half of the birds (7 birds in 2011, 6 birds in 2012 and 9 birds in 2014) were transferred to individual home cages facing northwest in an outdoor aviary with access to fresh air and to the natural celestial cues towards the NW. If sitting close to the mesh, the birds’ angle of view of the environment was up to 180° including the sunset point. The other half of the birds served as a control group, which was kept in the windowless animal room all the time.

### Spring experiments

During their first individual spring migration, the birds were tested again for their magnetic compass orientation response from March 15^th^ until April 11^th^ 2011, from March 12^th^ until April 12^th^ 2012 and March 26^th^ until April 9^th^ 2014, respectively, in grounded aluminium shielded wooden huts^46^,^47^, which screened electromagnetic disturbances up to 20 MHz by approximately two orders of magnitude and left the static geomagnetic field unaffected. The huts were illuminated by dim light bulbs covered by a plastic plane to produce diffuse homogeneous light conditions with an intensity of 2.5 mW/m^2^. The magnetic field was generated by a three-dimensional Merrit four-coil system with the current running anti-parallel through the double-wrapped coils^46^,^47^,^48^ simulating the local geomagnetic field with a total intensity of 48,880 nT ± 520 nT (s.d.), a declination of 0° and an inclination of 67.5° ± 0.4° (s.d.). Each night, in each hut, up to nine birds were simultaneously tested once or twice for one hour in Emlen funnel cages^49^ which were arranged on a wooden table in the centre of the coil system, equipped inside with scratch-sensitive paper^50^ and covered by an opaque plastic lid. Between tests, the experimental birds were continuously kept in the outdoor aviary and thus, had access to celestial cues throughout the spring. The control group of birds was kept in the windowless animal room between tests and thus, had no access to natural celestial cues at all. Experimental and control birds were tested simultaneously on most nights. This served to minimize any potential effects that weather could have on the orientation results^51^.

### Ringing data

As a reference direction for our orientation tests and for the associated *V* test, which takes the expected orientation direction into account^52^, we calculated the species-specific migratory directions of free-flying European robins from all same-season recovery data of wild European robins, which were either ringed or recovered in Lower Saxony (Germany) during autumn or spring migration, respectively.

**Figure 3:**
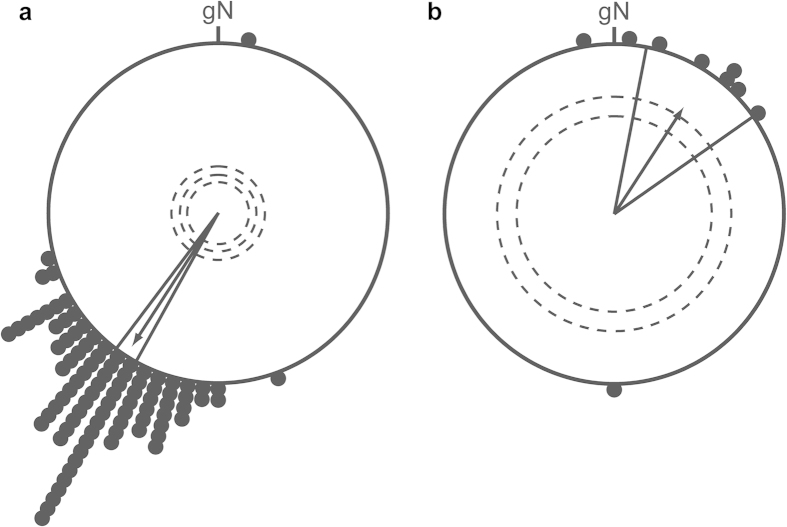
Mean orientation directions gained from ringing recovery data of European robins. (**a**) Autumn migration direction (mean = 213° ± 4°, r = 0.94, n = 90, *P* < 0.001). Birds were ringed in Lower Saxony (Germany) during the breeding season or during autumn and recovered during the same autumn on migration or at the wintering site. (**b**) Spring migration direction (mean = 33° ± 22°, r = 0.74, n = 9, *P* = 0.004). Birds were ringed in Lower Saxony during winter or during the following spring and were recovered during the same spring on migration or at the breeding site. Black dots denote individual loxodrome recovery directions relative to the ringing place (geographic North = gN). The arrows represent the group mean recovery directions. The inner dotted circles indicate, from centre to margins, the 5%, 1% or 0.1% significance levels, respectively, according to the Rayleigh test. The lines flanking the mean orientation vector indicate its 95% confidence intervals.

The ringing and recovery data from European robins ringed in Lower Saxony (Germany) were kindly provided by Olaf Geiter from the Institute of Avian Research, Wilhelmshaven (Germany), which is the northwest German ringing centre (http://www.ifv-vogelwarte.de). Only birds recovered more than 50 km away from the ringing place^53^ were included into the analysis. Pure same-autumn migration data were extracted in the following way: only data from birds that were ringed in Lower Saxony during the breeding period or during migration (mid-May until end of November) and recovered during autumn migration or at their wintering site (from date of ringing until the end of February) were included for calculating the autumn migratory direction (n = 90). Pure same-spring migration data were extracted in the following way: only data from birds that were ringed in Lower Saxony during winter or during the following spring migration (December until end of April) and recovered during the spring migration or breeding season (from date of ringing until the end of July, n = 9) were included in the calculation of the spring migratory direction. As birds most likely follow constant compass direction routes in central Europe^53^, we calculated the loxodrome directions between the ringing and recovery locations according to Imboden and Imboden^54^. Based on these results, we calculated the mean orientation angles for autumn and spring migration of wild, free-flying European robins passing through or breeding in Lower Saxony by vector addition. The mean recovery direction of European robins in autumn was 213° ± 4° (r = 0.94, n = 90, *P* < 0.001; Fig. 3a) and in spring 33° ± 22° (r = 0.74, n = 9, *P* = 0.004; Fig. 3b). These directions coincide well with the migration axis of the overall German population of European robins^55^ and also with the control directions we observed in orientation tests with wild-caught European robins^46^.

### Data analysis

From the distribution of the scratches on the paper, a mean orientation angle, activity level and concentration of the mean angle were recorded for each individual for each night by two researchers working independently from one another. When both orientation values deviated by more than 30°, the paper was re-analysed by a third person and eventually categorised as random if no agreement was possible. Only papers with unimodal orientation and with more than 100 scratches were included in the analysis^56^. Out of 547 individual spring orientation tests, we had to exclude 130 scratch papers due to too little activity, 12 scratch papers due to bimodality and 45 papers due to random orientation of the scratches. The individual orientation angles were analysed using Matlab R2008b. For each individual, a mean orientation vector over all tests was calculated by vector addition. For each condition, a group mean orientation vector was calculated by vector addition of unit vectors pointing in the mean directions of each of the individual birds. This group mean vector was tested for significance by the Rayleigh test^52^ and for directedness considering the expected species-specific seasonal migratory direction according to ringing recoveries by the *V* test^52^. We also tested whether the 95% confidence interval for the mean vector included the expected direction defined for the preceding *V* test^57^. The mean directions chosen by each group were tested for uniformity over the three test seasons by a Watson-Williams F test^52^,^58^. Additionally, we compared the concentrations (r-values) of the spring distributions between the experimental and control group by the bootstrap technique^59^,^60^. With this technique, a random sample of orientation directions (n = 19) is drawn with replacement from the sample of orientation angles present in the experimental group (n = 22). Based on these 19 orientation angles, the corresponding r-value is calculated^53^. This procedure is repeated 100,000 times. In the next step, the resulting 100,000 r-values are ranked in ascending order. The values at rank 500 and rank 99,550 define the 99% confidence limits for the actually observed r-value of the experimental group. If the actually observed r-value of the control group lies outside this 99% confidence interval, the concentration/directedness differs significantly by *P* < 0.01 between the experimental and control group. This procedure was also applied the other way around (a random sample of orientation directions (n = 22) was drawn with replacement from the sample of orientation angles present in the control group (n = 19)).

## Additional Information

**How to cite this article**: Alert, B. *et al.* Re-calibration of the magnetic compass in hand-raised European robins (*Erithacus rubecula*). *Sci. Rep.* **5**, 14323; doi: 10.1038/srep14323 (2015).

## Figures and Tables

**Table 1:** Orientation results of the birds of the experimental and control groups relative to magnetic north in the normal magnetic field without access to visual cues during their individual first autumn migratory season 2010, 2011 and 2013 and during the consecutive spring migratory seasons 2011, 2012 and 2014, respectively.

**Season**	**Birds**	**α**	**r**	**n**	***P***	***V***	***Pv***
autumn 2010	experimental	111°	0.19	7	0.796	−0.038	0.555
autumn 2010	control	25°	0.64	6	0.079	−0.637	0.988
autumn 2011	experimental	132°	0.28	6	0.647	0.044	0.442
autumn 2011	control	33°	0.36	7	0.421	−0.359	0.908
autumn 2013	experimental	328°	0.16	9	0.8	−0.069	0.612
autumn 2013	control	304°	0.38	6	0.439	−0.004	0.506
all autumns	experimental	102°	0.08	22	0.876	−0.028	0.574
all autumns	control	9°	0.37	19	0.076	−0.335	0.981
spring 2011	experimental	19°	0.55	7	0.122	0.531	0.022
spring 2011	control	23°	0.27	6	0.676	0.26	0.189
spring 2012	experimental	74°	0.295	6	0.613	0.223	0.226
spring 2012	control	268°	0.49	7	0.194	−0.282	0.85
spring 2014	experimental	77°	0.55	9	0.064	0.395	0.047
spring 2014	control	116°	0.27	6	0.663	0.032	0.457
all springs	experimental	56° ± 46°	0.43	22	0.017	0.391	0.004
all springs	control	294°	0.08	19	0.895	−0.012	0.529

Values are given for the group mean orientation angle α, group mean vector length r, number of tested individuals n and the resulting *P* value from the Rayleigh test. For the spring orientation data, the *V* and corresponding *Pv* values are given for the *V* test according to the expected orientation direction of 213° in autumn and 33° in spring.
